# Supplementary material for: Molecular details of ruthenium red pore block in TRPV channels
Source: EMBO Rep. 2024 Jan 15;25(2):8. doi: 10.1038/s44319-023-00050-0 (PMC10897480; doi:10.1038/s44319-023-00050-0)
Supplement: Supplementary file 1 — Appendix [file 44319_2023_50_MOESM1_ESM.pdf]

Appendix

Table of contents

**Appendix Table S1.** Cryo-EM data collection and model statistics	**2**
**Appendix Figure S1.** TRPV2 cryoEM data quality.	**3**
**Appendix Figure S2.** TRPV2 cryoEM processing trees.	**4**
**Appendix Figure S3.** $TRPV2_{2APB+RR}$ and $TRPV2_{RR}$ vanilloid pocket densities.	**5**
**Appendix Figure S4.** Half map density of $TRPV2_{2APB+RR}$ and $TRPV2_{RR}$.	**6**
**Appendix Figure S5.** TRPV5 cryoEM data quality.	**7**
**Appendix Figure S6.** TRPV5 with RR cryoEM processing trees.	**8**
**Appendix Figure S7.** TRPV5 cryoEM selectivity filter density without symmetry.	**9**
**Appendix Figure S8.** Half map density of $TRPV5_{RR}$ and $TRPV5_{PIP2+RR}$.	**10**
**Appendix Figure S9.** TRPV5 cryoEM PI(4,5)P_2 binding site and vanilloid pocket densities.	**11**
**Appendix Figure S10.** RR block comparison between TRPV5 and TRPV6.	**12**

Appendix Table S1. Cryo-EM data collection and model statistics

	**TRPV2$_{RR}$** (EMD-29046, PDB 8FFL)	**TRPV2$_{2APB+RR}$** (EMD-29047, PDB 8FFM)	**TRPV5$_{RR}$** (EMD-29048, PDB 8FFN)	**TRPV5$_{PIP2+RR}$** (EMD-29051, PDB 8FFQ)
**Data collection and processing**				
Magnification	81,000x	105,000x	105,000x	105,000x
Voltage (kV)	300	300	300	300
Camera	K3	K3	K3	K3
Electron exposure (e–/Å^2)			43	42.9
Defocus range (μm)	-0.5 to -2.0	-0.5 to -2.0	-0.5 to -2.0	-0.8 to -2.5
Pixel size (Å)	1.07	0.834	0.84	0.83
Micrographs	12,659	10,643	9,175	12,412
Particles from 2D classification	433,561	516,889	130,744	6,859,887
Final particles	55,445	65,741	39,976	368,030
Symmetry	C1	C4	C4	C4
Map resolution (Å)	3.47	2.9	2.96	2.65
FSC threshold	0.143	0.143	0.143	0.143
**Model Refinement**				
Model composition				
Nonhydrogen atoms	19,675	20,067	19,871	19,679
Protein residues	2,426	2,492	2,408	2,408
Ligands	4	9	17	13
B factors (Å^2)				
Protein	45.6	60.19	43.46	43.46
Ligand	32.33	80.01	42.10	35.39
R.M.S. deviations				
Bond lengths (Å)	0.002	0.011	0.025	0.028
Bond angles (°)	0.488	1.873	2.080	2.466
Validation				
MolProbity score	1.18	1.14	1.32	1.09
Clashscore	3.88	2.07	5.19	2.98
Rotamer outliers (%)	0.29	0.38	0.58	0.00
Ramachandran plot				
Favored (%)	98.94	97.05	97.82	98.49
Allowed (%)	1.06	2.95	2.18	1.51
Disallowed (%)	0.00	0.00	0.00	0.00

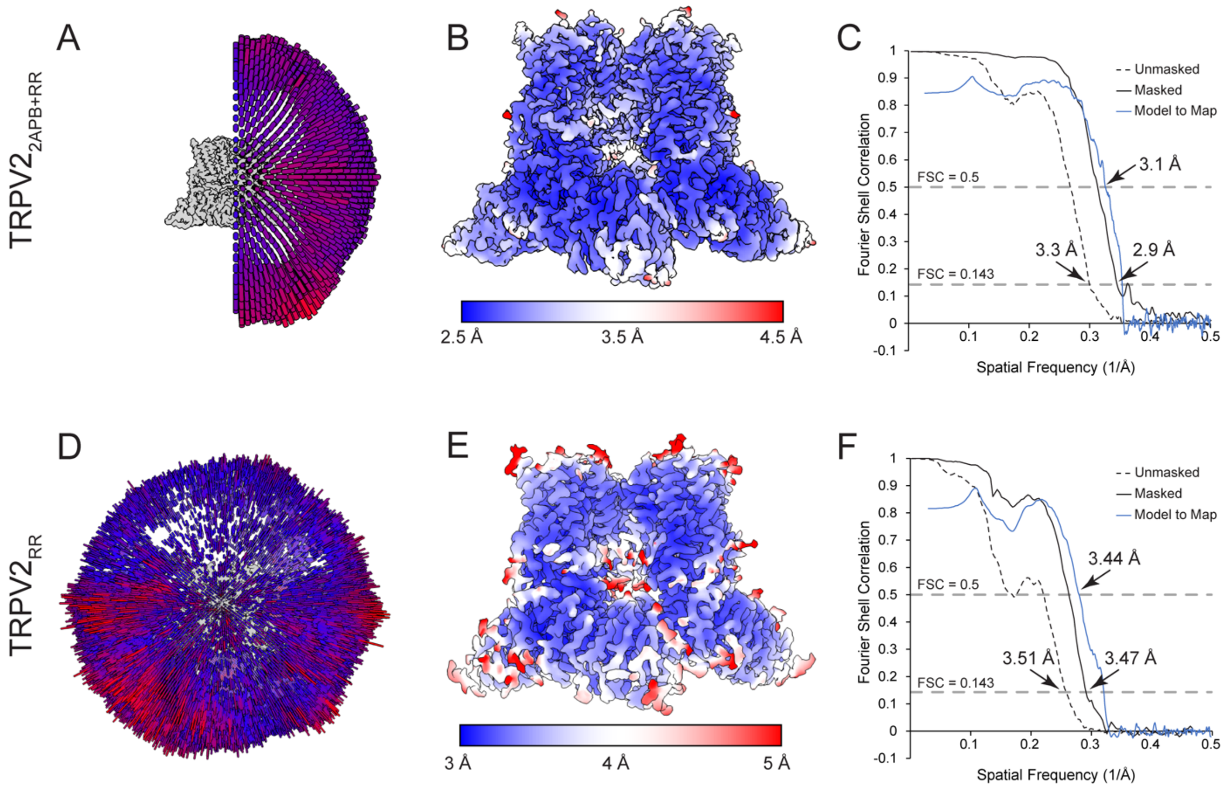

Appendix Figure S1. TRPV2 cryoEM data quality.

A Angular distribution of $TRPV2_{2APB+RR}$.

B Local resolution calculated using BlocRes of $TRPV2_{2APB+RR}$.

C The unmasked (dashed line), masked (black line), and model to map (blue line) FSCs curves of $TRPV2_{2APB+RR}$.

D Angular distribution of $TRPV2_{RR}$.

E Local resolution calculated using BlocRes of $TRPV2_{RR}$.

F The unmasked (dashed line), masked (black line), and model to map (blue line) FSCs curves of $TRPV2_{RR}$

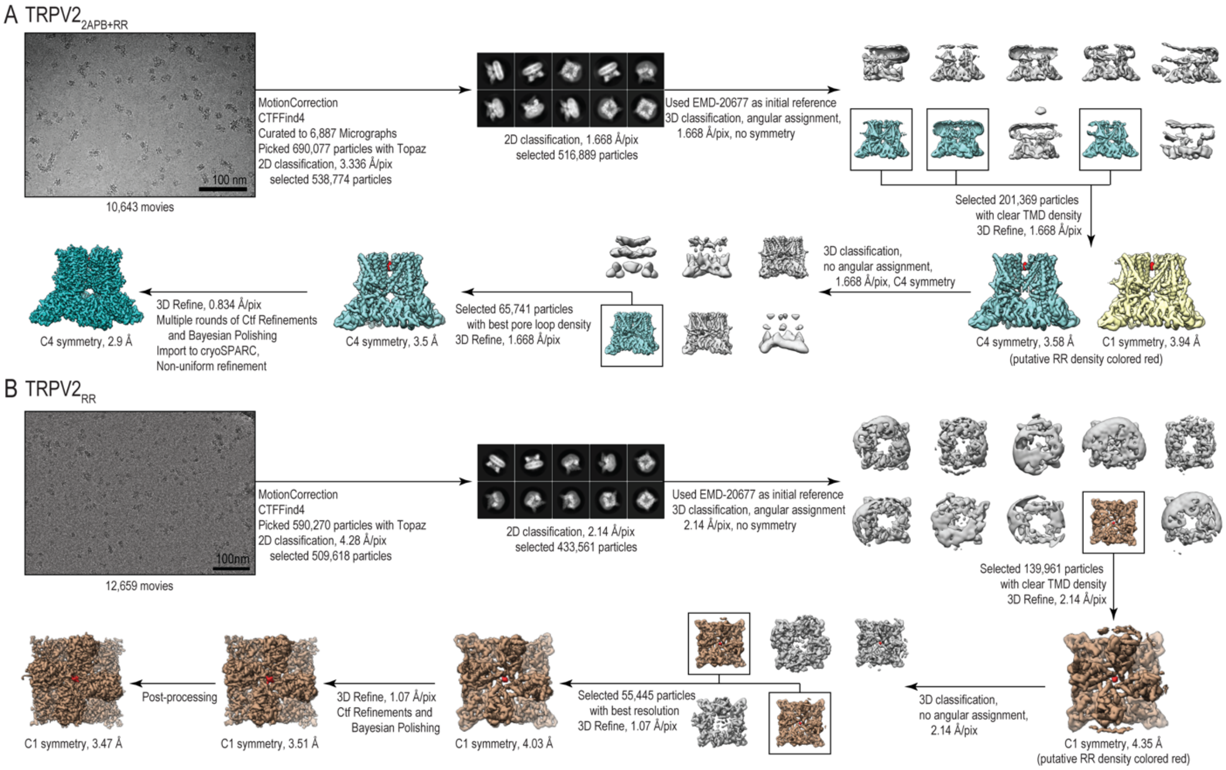

Appendix Figure S2. TRPV2 cryoEM processing trees.

A,B Processing trees for $TRPV2_{2APB+RR}$ **(A)** and $TRPV2_{RR}$ **(B)**.

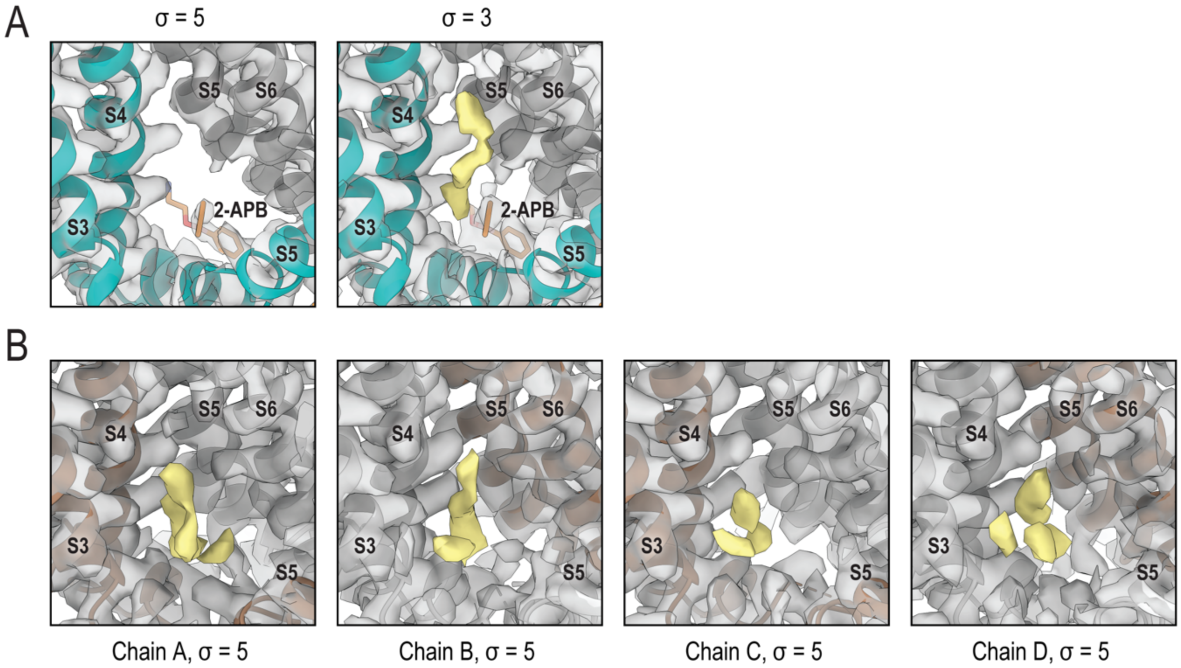

Appendix Figure S3. TRPV2$_{2APB+RR}$ and TRPV2$_{RR}$ vanilloid pocket densities.

A Map density and cartoon representation of the TRPV2$_{2APB+RR}$ vanilloid pocket. One monomer is colored teal, the adjacent monomers are colored grey. Putative lipid density is colored khaki, 2-APB is colored orange. The map is contoured at $\sigma = 5$ (left) or $\sigma = 3$ (right).

B Map density and cartoon representation of each TRPV2$_{RR}$ vanilloid pocket. Chains A and C are colored brown, Chains B and D are colored grey. The map is contoured at $\sigma = 5$. Putative lipid density is colored khaki.

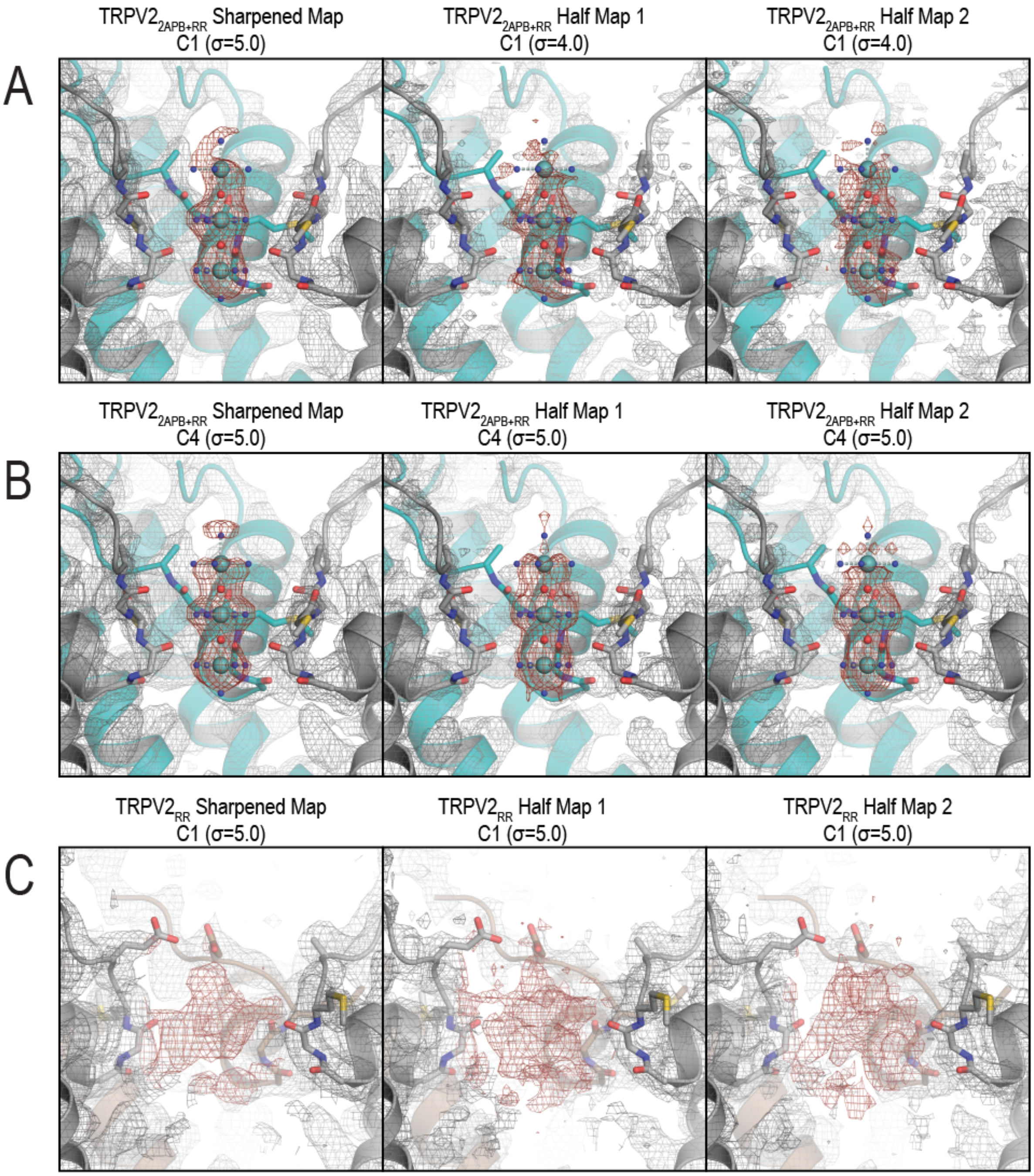

Appendix Figure S4. Half map density of TRPV2$_{2APB+RR}$ and TRPV2$_{RR}$.

A-C Main map and half map density for TRPV2$_{2APB+RR}$ refined without symmetry (**A**), TRPV2$_{2APB+RR}$ refined with C4 symmetry (**B**), and TRPV2$_{RR}$ refined without symmetry (**C**). Density for RR is red.

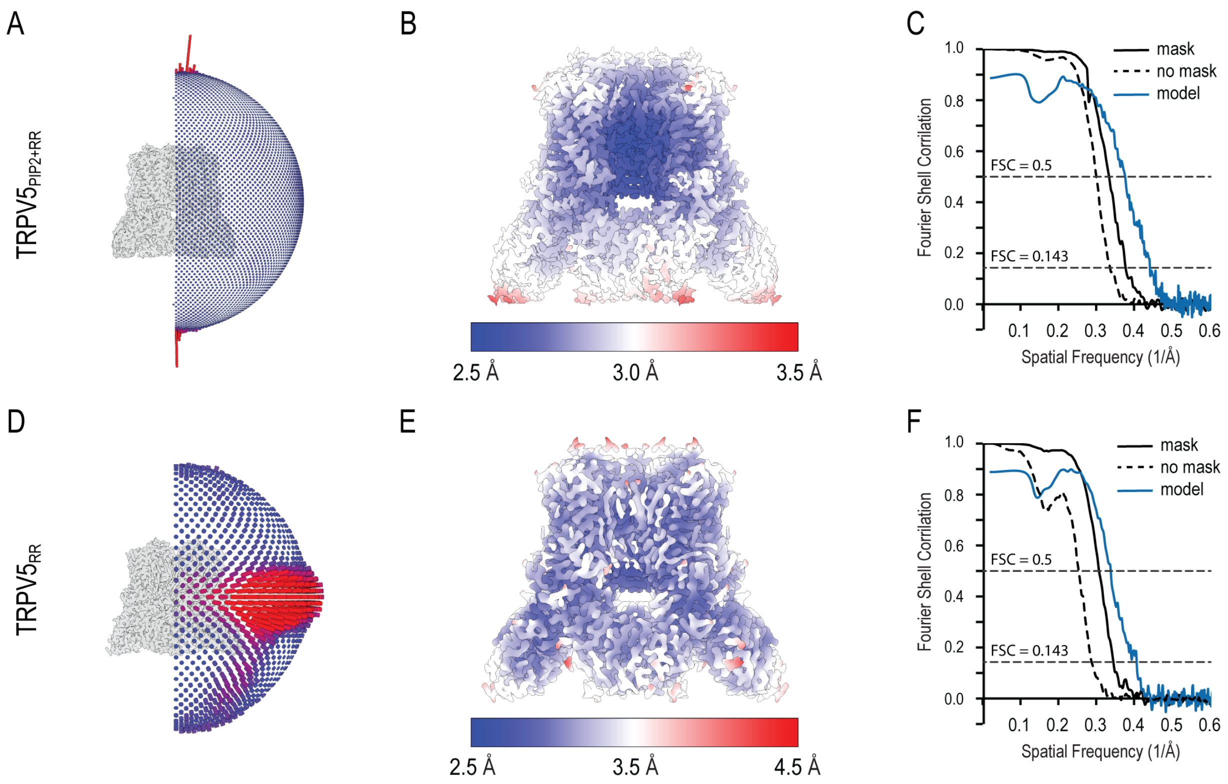

Appendix Figure S5. TRPV5 cryoEM data quality.

A Angular distribution of $TRPV5_{PIP2+RR}$.

B Local resolution calculated using BlocRes of $TRPV5_{PIP2+RR}$.

C The unmasked (dashed line), masked (black line), and model to map (blue line) FSCs curves of $TRPV5_{PIP2+RR}$.

D Angular distribution of $TRPV5_{RR}$.

E Local resolution calculated using BlocRes of $TRPV5_{RR}$.

F The unmasked (dashed line), masked (black line), and model to map (blue line) FSCs curves of $TRPV5_{RR}$.

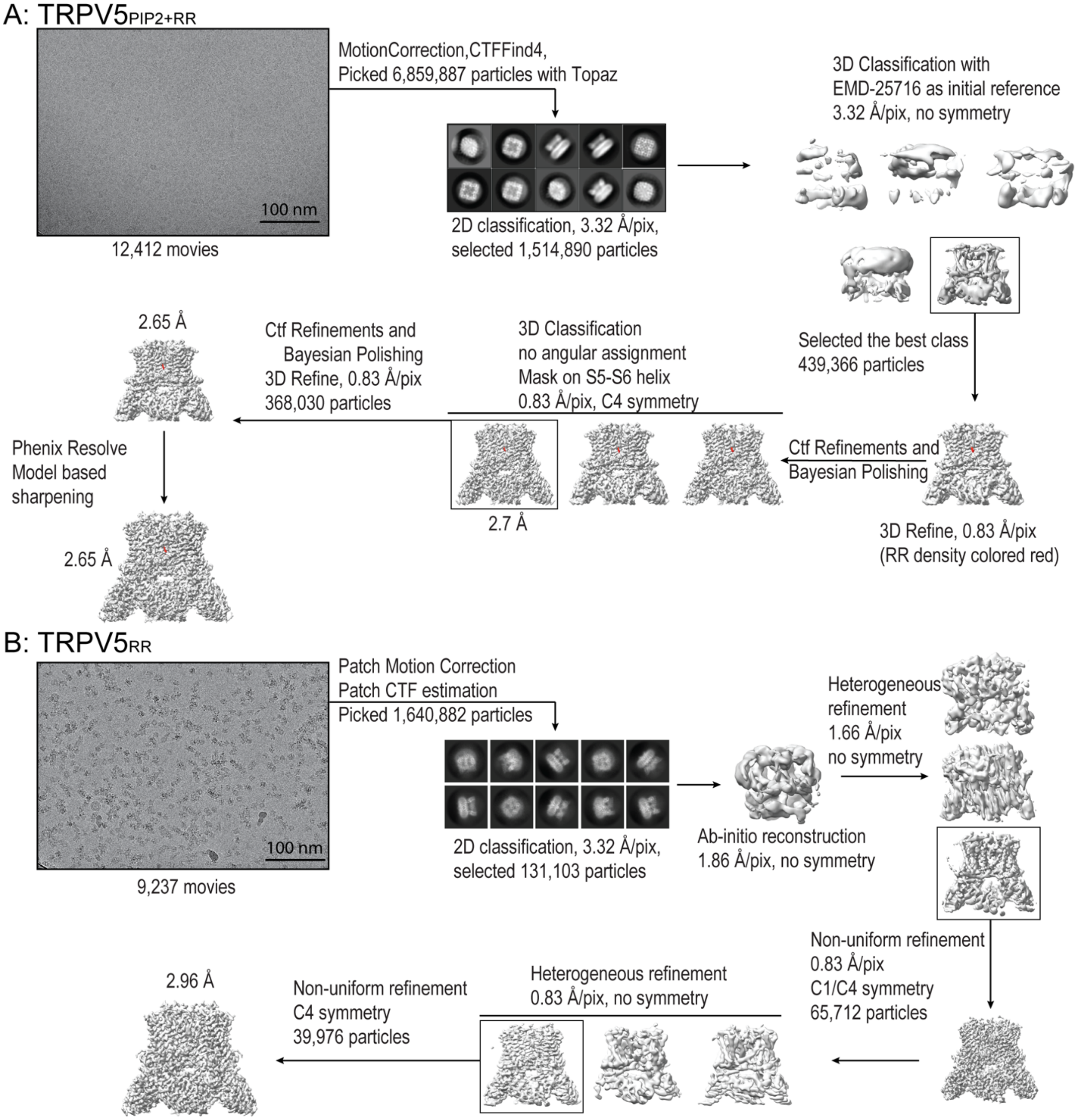

Appendix Figure S6. TRPV5 with RR cryoEM processing trees.
A,B Processing trees for $TRPV5_{PIP2+RR}$ (**A**) and $TRPV5_{RR}$ (**B**).

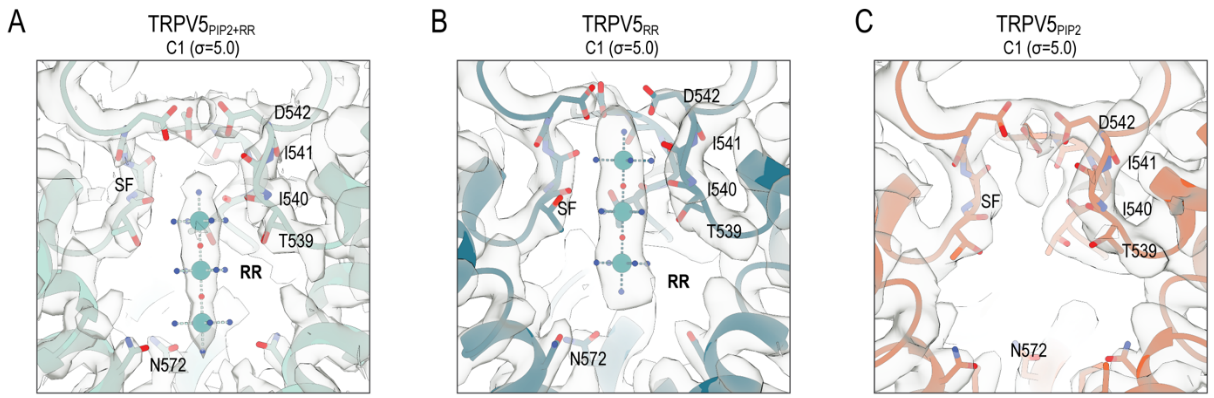

Appendix Figure S7. TRPV5 cryoEM selectivity filter density without symmetry.
A-C Map density at the Selectivity Filter for $TRPV5_{PIP2+RR}$ (**A**), $TRPV5_{RR}$ (**B**), and $TRPV5_{PIP2}$ (EMD-29049, PDB 8FFO) (**C**) refined without symmetry. Maps contoured at $\sigma = 5$.

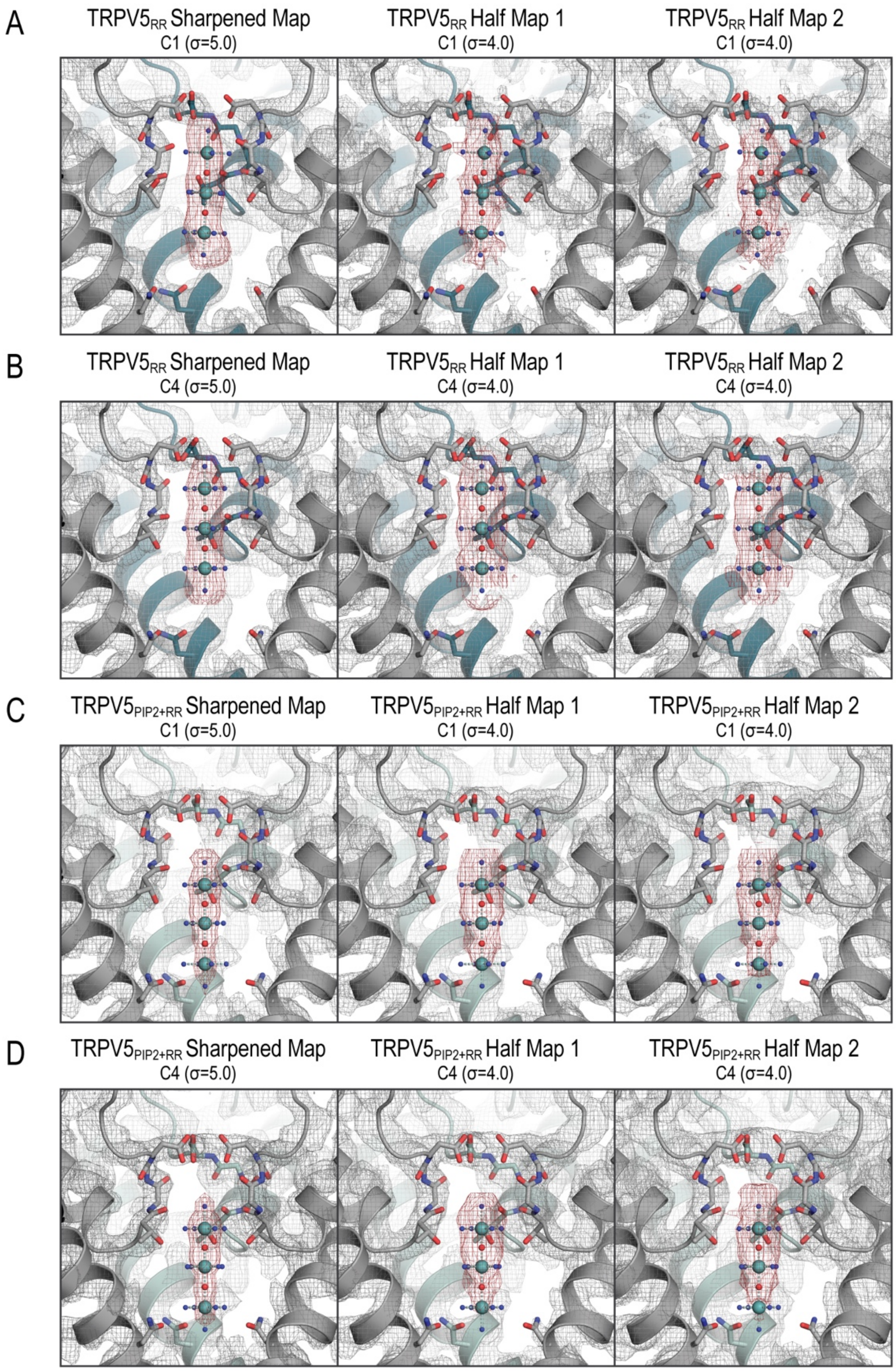

Appendix Figure S8. Half map density of $TRPV5_{RR}$ and $TRPV5_{PIP2+RR}$.
A-D Main map and half map density for $TRPV5_{RR}$ refined without symmetry (**A**), $TRPV5_{RR}$ refined with C4 symmetry (**B**), $TRPV5_{PIP2+RR}$ refined without symmetry (**C**), and $TRPV5_{PIP2+RR}$ refined with C4 symmetry (**D**). Density for RR is red.

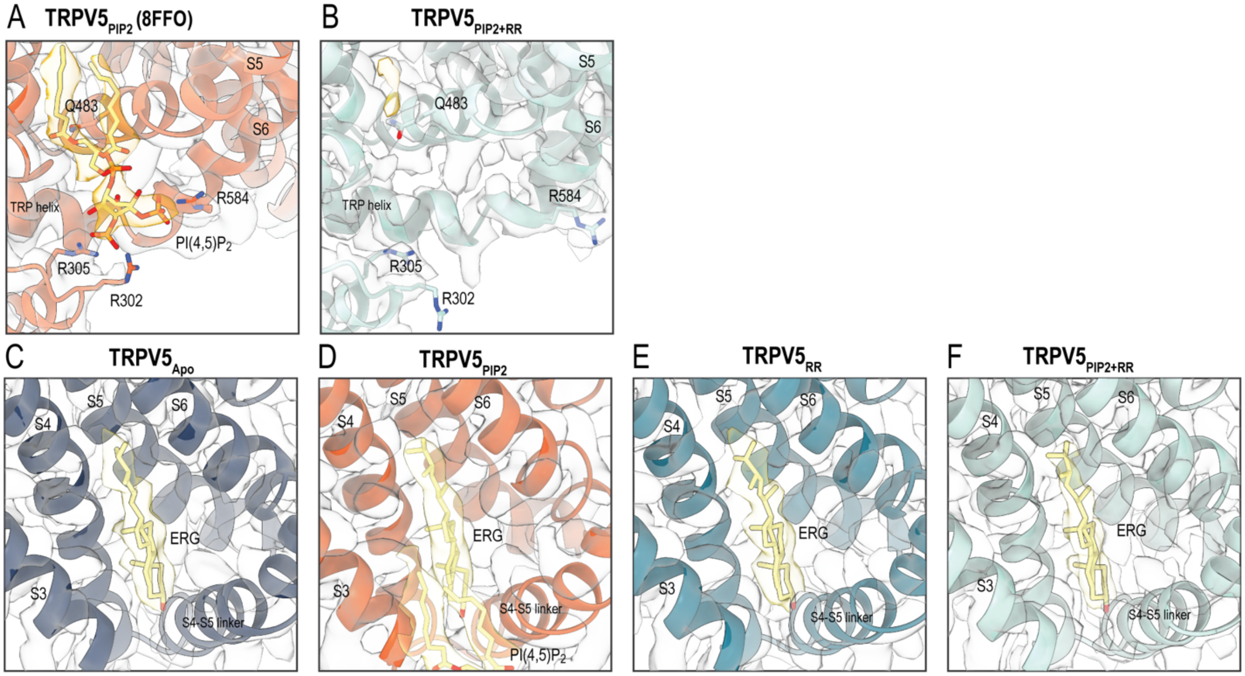

Appendix Figure S9. TRPV5 cryoEM PI(4,5)P_2 binding site and vanilloid pocket densities.
A,B Map density at the PI(4,5)P_2 binding site for $TRPV5_{PIP2}$ (EMD-29049, PDB 8FFO) (**A**) and $TRPV5_{PIP2+RR}$ (**B**).
C-F Ergosterol density at the vanilloid pocket for $TRPV5_{Apo}$ (**C**), $TRPV5_{PIP2}$ (**D**), $TRPV5_{RR}$ (**E**), and $TRPV5_{PIP2+RR}$ (**F**). Maps contoured at $\sigma = 3.0$.

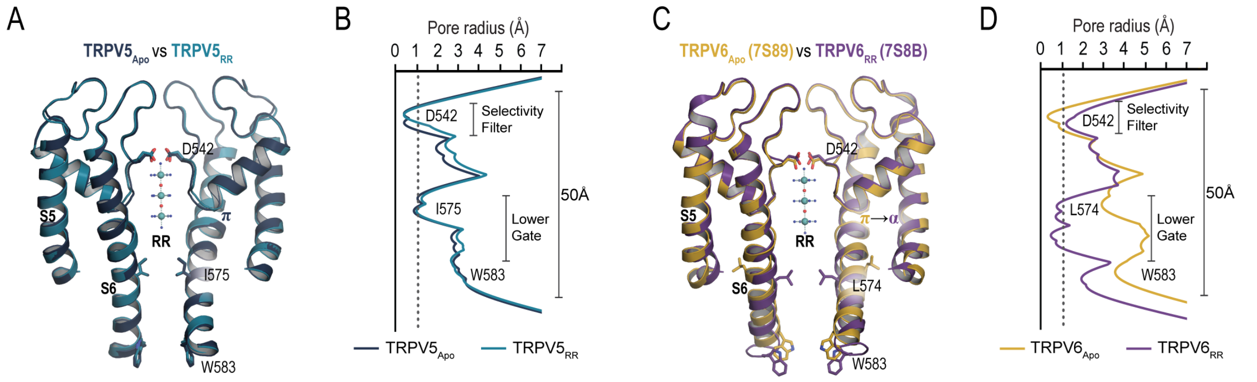

Appendix Figure S10. RR block comparison between TRPV5 and TRPV6.
A HOLE-generated pore profile of Apo and RR blocked TRPV5.
B Graphical representation of the pore profiles of $TRPV5_{Apo}$ (dark blue) and $TRPV5_{RR}$ (medium blue).
C HOLE-generated pore profile of Apo and RR blocked TRPV6.
D Graphical representation of the pore profiles of $TRPV6_{Apo}$ (gold) and $TRPV6_{RR}$ (purple). The radius of a dehydrated calcium ion is marked by a dotted gray line at 1.1 Å.
