# Supplementary material for: Molecular details of ruthenium red pore block in TRPV channels
Source: EMBO Rep. 2024 Jan 15;25(2):8. doi: 10.1038/s44319-023-00050-0 (PMC10897480; doi:10.1038/s44319-023-00050-0)
Supplement: Supplementary file 4 — Expanded View Figures [file 44319_2023_50_MOESM4_ESM.pdf]

Expanded View Figures

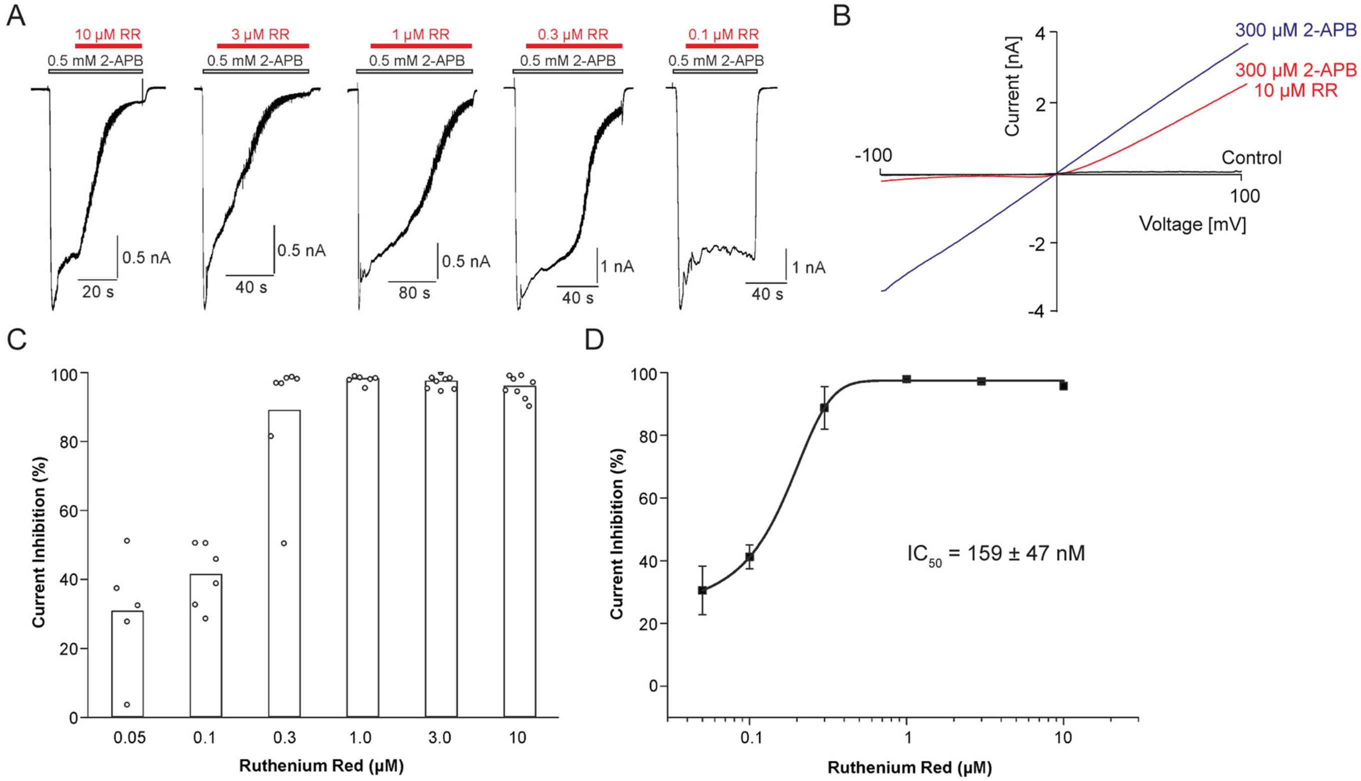

Figure EV1. Inhibition of 2-APB-induced TRPV2 currents by ruthenium red.

(**A**) Representative current traces from HEK293 cells displaying inhibition of 2-APB-induced currents by RR at different concentrations. RR was co-applied with 0.5 mM 2-APB once the current induced by 2-APB applied alone had reached steady state. Cells were held at −60 mV, and only one concentration of RR was tested on each cell examined. (**B**) Typical current trace on rTRPV2 recording during a 500 ms long voltage-ramp ranging from −100 to 100 mV. Note that RR-induced inhibition of 2-APB-induced currents was more effective on inward currents than on outward currents. (**C**) Bar columns displaying the average inhibition of inward currents evoked by 2-APB for each examined concentration of RR. ([RR] in nM, number of cells for each concentration): (0, 8), (50, 5), (100, 6), (300, 7), (1000, 6), (3000, 8) and (10,000, 8). (**D**) Dose-response curve for RR-induced inhibition of inward currents evoked by 2-APB on rTRPV2. Mean ± S.E.M. fractional block induced by each concentration of RR are given. The data were fitted with the Hill1 equation with the Origin software. Cells for each concentration are the same as (**C**): ([RR] in nM, number of cells for each concentration): (0, 8), (50, 5), (100, 6), (300, 7), (1000, 6), (3000, 8) and (10,000, 8). Source data are available online for this figure.

 © The Author(s)

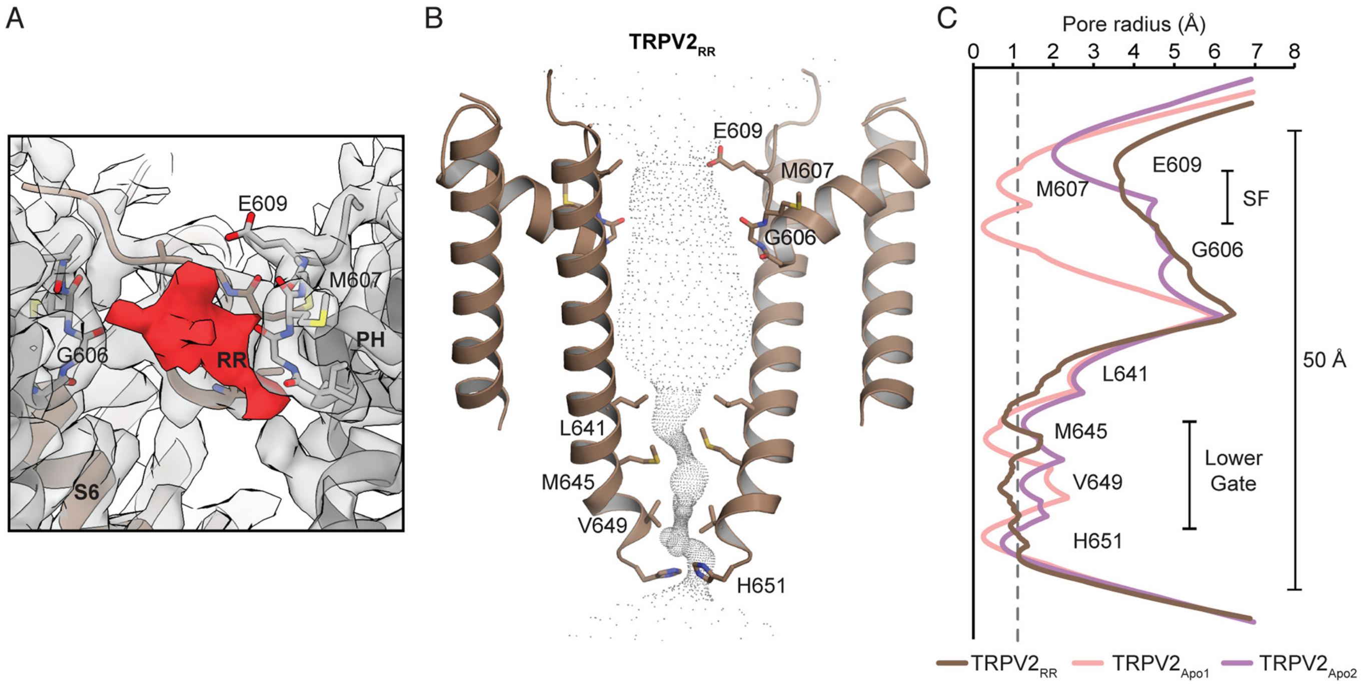

Figure EV2. Selectivity filter and pore of TRPV2$_{RR}$.

(**A**) Map density and cartoon representation of the possible RR binding site in TRPV2$_{RR}$. One monomer is colored brown, the adjacent monomers are colored gray. Putative density for RR is colored red. The map is contoured at $\sigma = 5$. (**B**) HOLE-generated pore profile of TRPV2$_{RR}$. (**C**) Graphical representation of the pore profiles of TRPV2$_{RR}$ (brown), TRPV2$_{Apo1}$ (PDB 6U84, pink) and TRPV2$_{Apo2}$ (PDB 6U86, purple). The radius of a dehydrated calcium ion is marked by a dotted gray line at 1.1 Å.

© The Author(s)

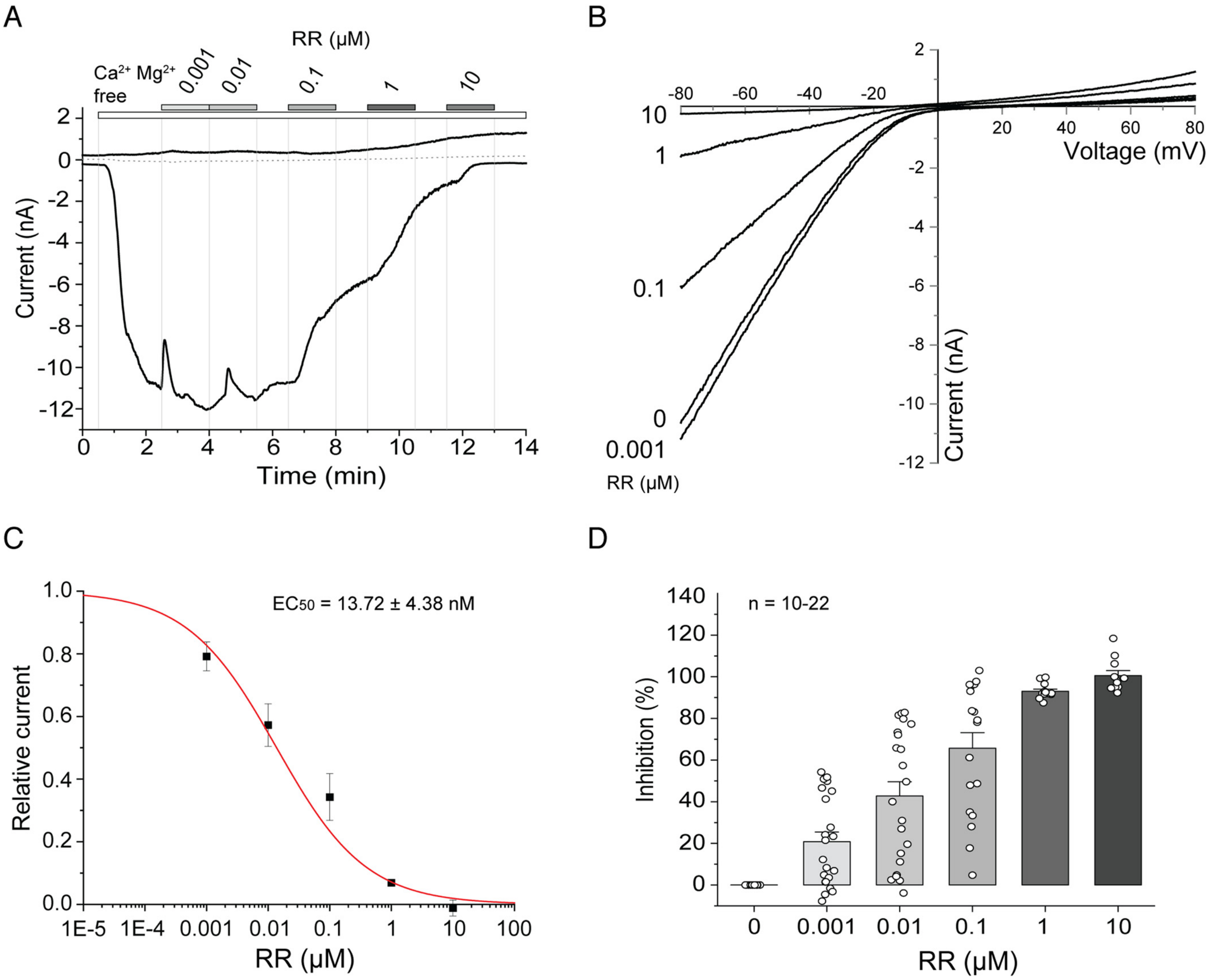

Figure EV3. Inhibition of TRPV5 currents by Ruthenium red.

(**A**) Representative current traces from HEK293 cells transfected with TRPV5, at 80 mV (upper trace) and −80 mV (lower trace). Monovalent currents were initiated by the application of a Ca^{2+} and Mg^{2+} free solution, as described in the methods section. Dotted line shows zero current, and applications of various concentrations of RR are shown with horizontal bars. (**B**) Representative ramp current–voltage (*I–V*) traces from (**A**) revealed characteristic inwardly rectifying TRPV5 currents in the absence or presence of different concentrations of RR. (**C**) Relative current levels at −80 mV after application of the various concentrations of RR. The data were fitted using the Hill1 equation with the Origin2021 software and plotted as mean ± SEM. ([RR] in nM, number of cells for each concentration): (0, 22), (1, 22), (10, 22), (100, 18), (1000, 12), and (10,000, 10). (**D**) Bar graph (mean ± SEM and individual values) of inhibition of TRPV5-mediated monovalent currents by various concentrations of RR. Cells for each concentration are the same as (**C**): ([RR] in nM, number of cells for each concentration): (0, 22), (1, 22), (10, 22), (100, 18), (1000, 12), and (10,000, 10). Source data are available online for this figure.

 © The Author(s)

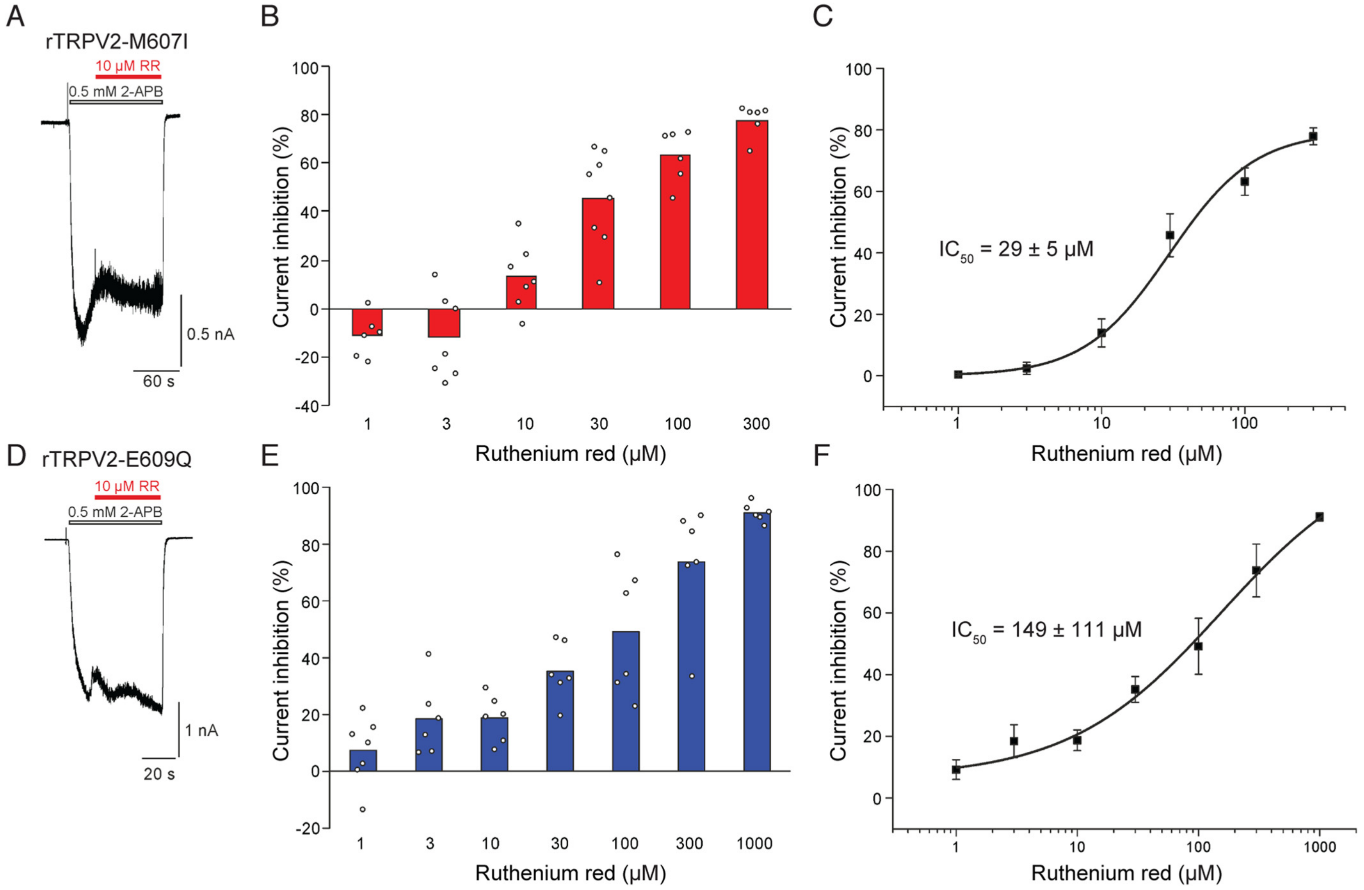

Figure EV4. Mutations to the TRPV2 selectivity filter reduce RR pore block.

(**A**) Representative current trace displaying inhibition of 2-APB-induced current by 10 μM RR on the mutant rTRPV2-M607I from HEK293 cells. (**B**) RR was co-applied with 0.5 mM 2-APB once the current induced by 2-APB applied alone had reached steady state. HEK293 Cells were held at −60 mV. (**B**, **E**) Bar columns displaying the average inhibition of inward currents evoked by 2-APB for each examined concentration of RR on rTRPV2-M607I. ([RR] in μM, number of cells for each concentration): (0, 8), (1, 6), (3, 7), (10, 7), (30, 8), (100, 6) and (300, 6). (**C**) Dose–response curve (mean ± SEM) for RR-induced inhibition of inward currents evoked by 2-APB on rTRPV2-M607I. Cells for each concentration are the same as (**B**): ([RR] in μM, number of cells for each concentration): (0, 8), (1, 6), (3, 7), (10, 7), (30, 8), (100, 6) and (300, 6). (**D**) Representative current trace displaying inhibition of 2-APB-induced currents by 10 μM RR on the mutant rTRPV2-E609Q. (**E**) RR was co-applied with 0.5 mM 2-APB once the current induced by 2-APB applied alone had reached steady state. HEK293 Cells were held at −60 mV. (**B**, **E**) Bar columns displaying the average inhibition of inward currents evoked by 2-APB for each examined concentration of RR on rTRPV2-E609Q. $n = 6$–8 cells for each concentration. ([RR] in μM, number of cells for each concentration): (0, 7), (1, 7), (3, 6), (10, 6), (30, 6), (100, 6), (300, 6), and (1000, 6). (**F**) Dose–response curves for RR-induced inhibition of inward currents evoked by 2-APB on rTRPV2-E609Q. Mean ± S.E.M. fractional block induced by each concentration of RR are given. The data were fitted with the Hill1 equation with the Origin software. Cells for each concentration are the same as (**F**): ([RR] in μM, number of cells for each concentration): (0, 7), (1, 7), (3, 6), (10, 6), (30, 6), (100, 6), (300, 6), and (1000, 6). Source data are available online for this figure.

© The Author(s)

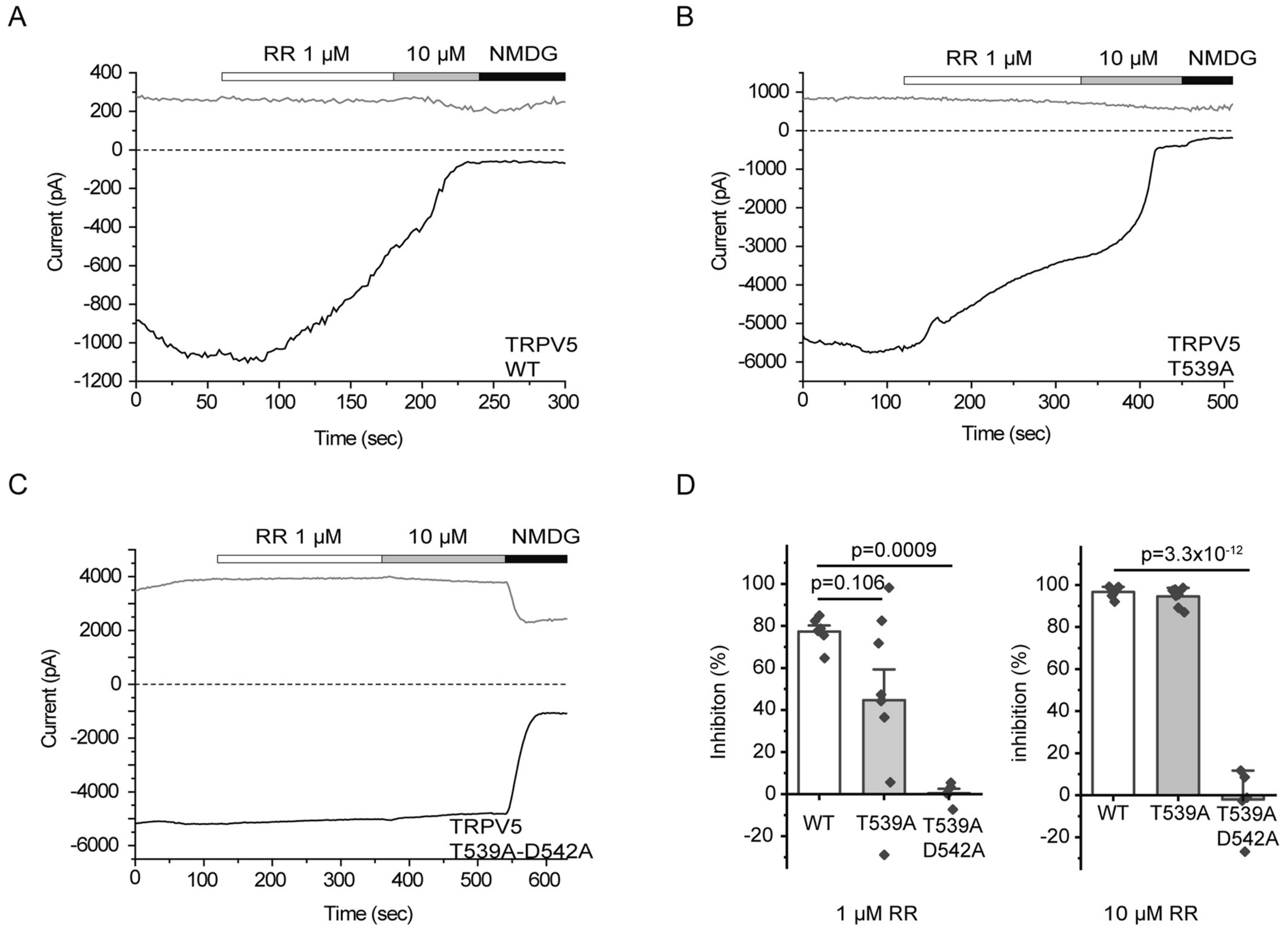

Figure EV5. Mutations to the TRPV5 selectivity filter reduce RR pore block.

(**A–C**) Representative trace for TRPV5 WT (**A**), T539A (**B**), and T539A/D542A (**C**) currents at 80 mV (upper trace) and −80 mV (lower trace). The application of a Ca^{2+} and Mg^{2+} free solution was used as a bath solution (extracellular solution) to initiate TRPV5 currents. The dotted line shows zero current, and applications of 1 or 10 μM of RR are shown with horizontal bars. At the last minute of recording, NMDG was applied to inhibit ion permeability through the TRPV5 pore. (**D**) Bar graph (mean ± SEM and individual values) of inhibition of WT or mutant TRPV5 currents by 1 or 10 μM RR. $n = 6$ cells transfected with WT TRPV5 for 1 or 10 μM RR, $n = 8$ for T539A, and $n = 5$ for T539A + D542A. Statistical significance was calculated with one-way analysis of variance, with Bonferroni's post hoc test. Source data are available online for this figure.

 © The Author(s)
